# Integrated Topical Skincare Enhances Picosecond Laser Treatment Outcomes for Photoaging: A Prospective Split-Face Trial

**DOI:** 10.1007/s00266-026-05993-y

**Published:** 2026-07-23

**Authors:** Liang-Chen Lin, Ya-Ting Yu, Hsuan-Jung Hsiao, Yun-Hsin Lai, Ching-Ting Pan, Yen-Jen Wang

**Affiliations:** 1 Bright Skin Clinic, HsinChu City, Taiwan; 2 L’Oréal Taiwan Co., Ltd., Taipei City, Taiwan; 3 Department of Dermatology, MacKay Memorial Hospital, Taipei City, Taiwan; 4 Department of Cosmetic Applications and Management, MacKay Junior College of Medicine, Nursing, and Management, New Taipei City, Taiwan

**Keywords:** Photoaging, Picosecond laser, Integrated skincare, Skin rejuvenation, Facial wrinkles, Randomized controlled trial

## Abstract

**Background:**

The 755-nm picosecond alexandrite laser with a diffractive lens array effectively treats photoaging in Asian patients but may cause transient adverse effects due to temporary disruption of the skin barrier.

**Objective:**

This study evaluated the efficacy and safety of a topical integrated skincare regimen containing CE Ferulic serum, Discoloration Defense (3% tranexamic acid), and Triple Lipid Restore 2:4:2 (CE/DD/242) following treatment with a 755-nm picosecond alexandrite laser with a diffractive lens array (DLA) in an Asian population.

**Methods:**

This single-center, randomized, evaluator-blinded, split-face controlled study enrolled 35 patients aged 25–63 years with facial photoaging. Each patient underwent three laser treatment sessions at four-week intervals. Patients applied Phyto Corrective serum and sunscreen to one cheek (control side) and adjunctive CE/DD/242 to the other cheek (treatment side). Clinical efficacy was evaluated using the VISIA® Complexion Analysis System, the FocuSkin® Skin Analyzer, and a participant satisfaction questionnaire. Self-reported redness or swelling was recorded by telephone follow-up.

**Results:**

Laser treatment significantly improved pores (p=0.0011), brown spots (p=0.0003), and skin moisture (p=0.0001). Adjunctive integrated skincare further enhanced wrinkle reduction (adjusted difference − 2.33, p=0.018) and texture improvement (adjusted difference − 0.63, p=0.027). Females over 46 years demonstrated greater wrinkle reduction (p=0.0437), while males showed greater elasticity improvement (p=0.0003). Temporary erythema occurred in 13 of 35 patients, while 18 reported no adverse effects. Patient satisfaction scores were higher on the CE/DD/242 treatment side.

**Conclusions:**

The 755-nm picosecond alexandrite laser with a diffractive lens array improves photoaging and overall skin quality. Adjunctive integrated skincare (CE/DD/242) provides synergistic clinical benefits with minimal adverse effects, resulting in greater improvements in wrinkles and skin texture.

**Level of Evidence I:**

Evidence obtained from at least one properly designed randomized controlled trial. This journal requires that authors assign a level of evidence to each article. For a full description of these Evidence-Based Medicine ratings, please refer to the Table of Contents or the online Instructions to Authors www.springer.com/00266.

**Supplementary Information:**

The online version contains supplementary material available at https://doi.org/10.1007/s00266-026-05993-y.

## Introduction

Energy‐based devices improve photoaging signs in darker skin yet leave notable gaps in treatment design and implementation [1, 2]. Studies report that methods using radiofrequency, 755-nm picosecond alexandrite laser with the diffractive lens array (DLA), intense pulsed light, combination approaches, and microfocused ultrasound yield measurable benefits—improved skin texture and skin laxity, reduced fine lines and wrinkles, and decreased pigmentation and pore size. [3–7] To achieve satisfactory result, multiple treatment sessions are often indicated. Patients undergoing picosecond laser with DLA treatment may experience skin sensitivity, transient compromised barrier function, increased transepidermal water loss (TEWL), and oxidative stress post-treatment. Laser-generated reactive oxygen species (ROS) affect human dermal fibroblasts by altering mitochondrial dynamics, which in turn activate downstream cascades such as ERK signaling and modulate cellular proliferation, motility, and viability [8]. The molecular mechanism of laser-induced oxidative stress involves initial mitochondrial ROS generation followed by cytosolic spread, accompanied by calcium spikes and membrane potential changes that can compromise cell viability at higher exposure levels [9–11]. Post-laser care therefore requires a multifaceted integrated approach, including removing oxidative stress, restoring the compromised skin barrier function, stabilizing activated melanocytes, reducing TEWL, and ensuring strict sun protection [12]. Inadequate post-laser care prolongs recovery and raises the risk of hyperpigmentation or recurrence. [13]

In vivo studies indicate alpha-tocopherol and ascorbic acid (vitamin C) have strong antioxidative properties [14]. Ferulic acid (FA) performed similarly to vitamin C in reducing signs of photoaging, improving skin hydration, elasticity, and pigmentation in comparative studies [15]. While FA tends to oxidize rapidly, presenting challenges in formulation stability [16], it demonstrates synergistic effects when combined with vitamin C and E, enhancing photoprotection and stability [17, 18]. The topical application of a vitamin C, vitamin E, and ferulic acid serum immediately following fractional ablative CO_2_ laser therapy did not result in associated complications [19]. Shi. et al. also showed incorporating CE Ferulic plus resveratrol BE serum following ablative fractional CO_2_ laser treatment reduced erythema and hyperpigmentation, promoting wound healing, and maintaining skin hydration [20]. A perioperative integrated skincare consensus was provided for noninvasive energy-based device (EBD) aesthetic procedures in clinical practice in China [12]. It was recommended to apply a plant-derived essence containing rosemary, olive leaf, mulberry root, and cucumber to the entire treated area to prevent allergic reactions to the compounded lidocaine cream before the EBD treatment and a 15% L-ascorbic acid repair essence immediately following the EBD treatment [12]. Phyto Corrective is a topical serum formulated with hyaluronic acid and botanical components including cucumber, thyme, and olive leaf extracts. It exerts beneficial effects on the skin through its combination of anti-inflammatory botanicals, hydrating hyaluronic acid, and antioxidant-rich extracts. These botanical antioxidants scavenge reactive oxygen species (ROS) and inhibit lipid peroxidation, thus mitigating oxidative damage to keratinocytes and dermal fibroblast following the EBD treatment [21]. Its use is particularly indicated for sensitive, post-procedural, or acne-prone skin. Cucumber extract possesses soothing and cooling effects, making it beneficial for reducing erythema and discomfort in post-laser skin conditions [22]. On the other hand, sebaceous glands’ function declines with age and cannot convert squalene to sterols like cholesterol. Skin-identical lipids (cholesterol, ceramide, and fatty acids) applied topically deposit in the stratum corneum reduce symptoms of compromised skin including dryness, scaling, redness, and itching after EBD treatment [23]. In some patients undergoing EBD treatments, increased prostaglandins and leukotrienes post-laser result in elevated activity of melanocytes and immune cells, which may increase risk of post-inflammatory hyperpigmentation (PIH) [24, 25]. We previously showed pre-treatment presence of activated melanocytes shown by optical coherence tomography predicted less melasma improvement after 755-nm picosecond alexandrite laser with DLA treatment [25]. Topical tranexamic acid (TXA), a potent plasmin inhibitor, is proposed to control pigmentation by inhibiting the release of inflammatory mediators involved in triggering melanogenesis [26]. It shows variable effectiveness in preventing and treating PIH across different skin types when used with EBD treatments [26–29]. Niacinamide inhibits melanosome transfer to keratinocytes and may be combined with TXA. The topical facial serum containing 3% TXA, 1% kojic acid, and 5% niacinamide has yielded a significant improvement in the appearance of PIH, skin texture, and skin tone homogeneity beginning at week 2 and continued through week 12 in patients with Fitzpatrick skin types I-IV [30]. In skin of color, pre-procedure and follow-up skincare with niacinamide, kojic acid, azelaic acid, and TXA-containing skincare is recommended to optimize energy treatment outcomes [31]. However, in practical applications, patients often believe they should wait at least one to two weeks before resuming their full regular skincare routine, particularly avoiding active ingredients such as vitamin C during this period.

Current research does not have enough data to confirm if combined cosmeceutical and picosecond laser treatments are more effective than standard methods for improving skin tone. It has been demonstrated that combining bioactive polymer dots with picosecond laser-induced optical breakdown (LIOB) enhances tissue regeneration [32]. Optical coherence tomography confirmed the formation of cavitation bubbles at the dermal–epidermal junction following LIOB, potentially providing dermal and epidermal repair. [32] We assessed the efficacy and safety of a 755-nm picosecond alexandrite laser with a DLA alongside integrated skincare products containing ferulic acid, TXA, and skin-identical lipids for facial photoaging, to standardize post-picosecond laser care protocols.

## Materials and Methods

### Study Design and Patients

This single-center, randomized, evaluator-blinded, split-face controlled study enrolled 35 patients aged 25–63 years with facial photoaging and was conducted at Bright Skin Clinic, HsinChu City, Taiwan. Patients aged 25–65 years with Fitzpatrick skin type II–V with symmetric photoaging signs were eligible to participate and received picosecond laser with a DLA treatment. Patients were excluded if they met any of the following criteria: (1) women who are pregnant, breast feeding, or plan to be pregnant in the following 1-year (2) patients with melasma; (3) patients who are taking oral contraceptive pills; (4) active infection or eczema on the face; (5) patients with autoimmune disease, history of photosensitivity, endocrine disease, or active liver disease; (6) patient who applied bleaching agents, steroid, or photosensitive medication within 3 months and who received energy-based devices treatment within 1 year; and (7) patient who have known allergy to any skincare product in this study. Written informed consent was signed and obtained from each patient. This study was approved by the Institutional Review Board Ethical Committee of Landseed International Hospital (IRB-24-044-A2)

### Randomization and Blinding

Each patient’s left and right cheeks were assigned to experimental and control groups in a 1:1 ratio using a random number table.

### Interventions

**Fig. 1:**
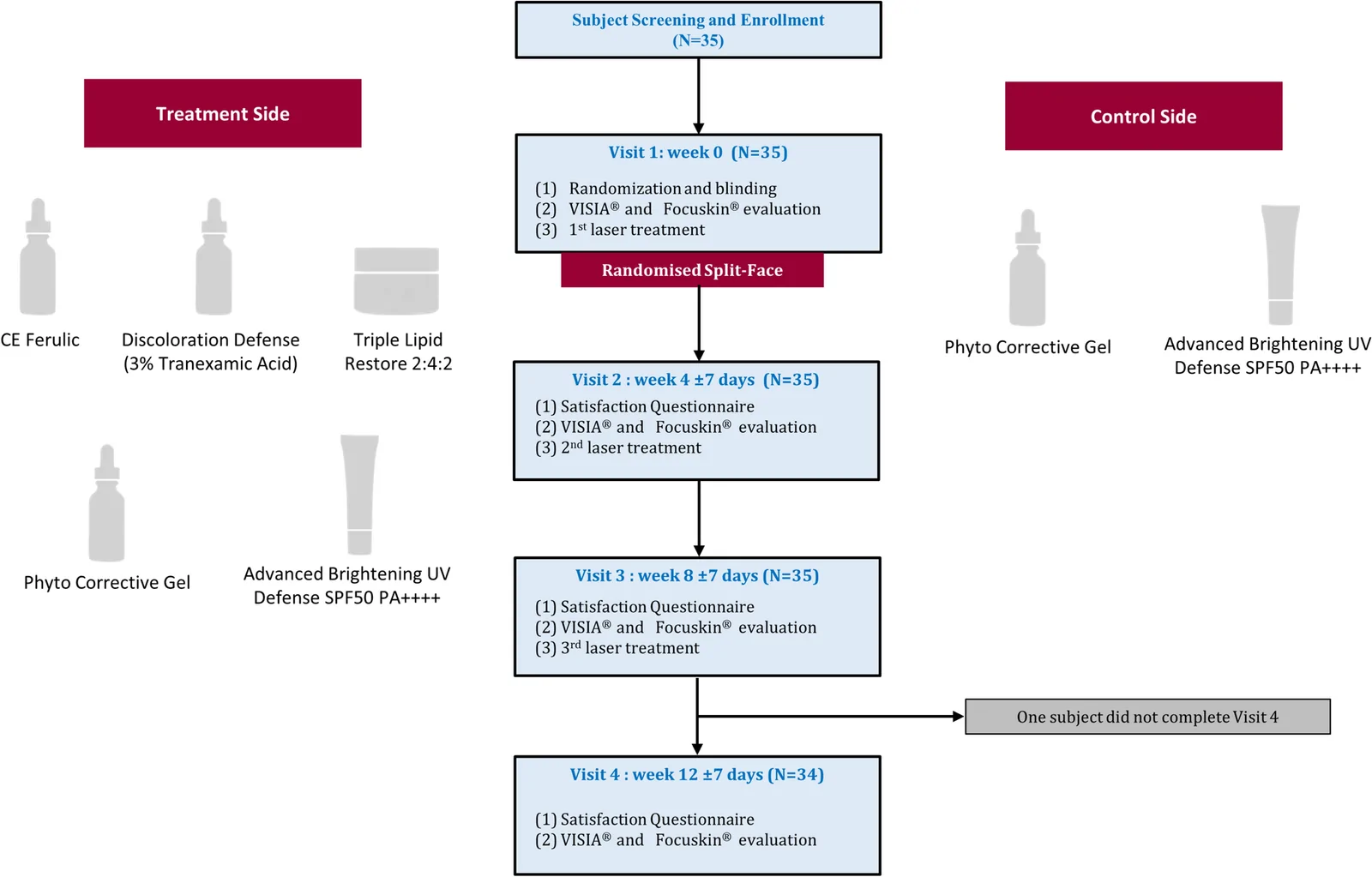
Schematic of study design. Each patient underwent three 755-nm picosecond alexandrite laser with a diffractive lens array (DLA) treatment on the whole face at 4-week interval. The patient had VISIA^®^ and FocuSkin^®^ evaluation before the laser treatment. This is a randomized split-face study: The patients applied Phyto Corrective and sunscreen to one cheek (control group) and an adjunctive integrated skincare composed of CE Ferulic Serum, Discoloration Defense (3% tranexamic acid), and Triple Lipid Restore 2:4:2 to the other cheek (treatment group)

All the patients underwent three 755-nm picosecond alexandrite laser sessions with diffractive lens array (DLA) (PicoSure® Pro 755, Cynosure, MA, USA) across the entire face at 1-month intervals (Fig. 1).

The energy settings consisted of a fluence range of 0.35 to 0.4 J/cm^2^, using an 8-mm spot size, 10 Hz frequency, and a 750-picosecond pulse duration. Due to the DLA, the high-energy zones (microbeams, representing 10% of the treated tissue) received a fluence of approximately 2.8 J/cm^2^, while the remaining 90% of the tissue (the low fluence background) was exposed to only 0.13 J/cm^2^. This method of energy delivery is particularly suitable for darker skin types. The 8-mm spot size produced roughly 230 microbeams, with the number of microbeams increasing as the spot size grows, though the fluence per microbeam decreases as the energy is distributed over a larger area. Consequently, an 8-mm spot size was selected instead of a smaller 6-mm spot size. Each session entailed two treatment cycles, delivering a total of approximately 1,800 to 2,500 pulses [33]. After 755-nm picosecond alexandrite laser with DLA treatment, CE Ferulic serum on the treatment side cheek and Phyto Corrective (serum) 5-8 drops was applied to the whole face immediately, followed by ice-cooling for 15 min.

### Home Care

Patients were subsequently advised to use a fragrance and soap­free cleanser with a near-physiological skin pH (< 6.5) and apply Phyto Corrective (serum) 5-8 drops twice daily (morning and evening), along with SPF50 PA++++ Advanced Brightening UV Defense sunscreen over the entire face using a teaspoon amount twice daily, in the morning and at noon, as their home care regimen. Patients were instructed to apply Phyto Corrective (serum) and sunscreen on the control side cheek. On the treatment side cheek, patients were instructed to apply an adjunctive integrated skincare composed of CE Ferulic serum 3–5 drops, Discoloration Defense serum 3–5 drops, and Triple Lipid Restore cream (CE/DD/242) half teaspoon twice daily (morning and evening) along with Phyto Corrective (serum) and sunscreen twice daily.

Phyto Corrective contains hyaluronic acid, thyme, olive leaf extract, cucumber extract, and rosemary leaf extract. CE Ferulic serum contains 0.5% ferulic acid, 15% ascorbic acid, and 1% tocopherol (Vit E). Discoloration Defense contains 3% tranexamic acid (TXA), 1% kojic acid, and 5% niacinamide (vitamin B3). Triple Lipid Restore cream 2:4:2 contains 2% ceramide, 4% cholesterol, and 2% fatty acids (Fig. 1).

### Postintervention Assessments

Objective facial skin analysis was performed using the VISIA® Complexion Analysis System (Canfield Scientific, NJ, USA) and the FocuSkin® Skin Analyzer (PIE Co., Ltd., South Korea). The VISIA® system has undergone independent evaluation demonstrating acceptable reproducibility and measurement precision under standardized imaging protocols. [34, 35]

All VISIA® images were obtained using a standardized capture protocol, including fixed chin positioning, consistent facial orientation, calibrated lighting, and identical camera settings across visits. Assessments were conducted at weeks 4, 8, and 12, thereby avoiding the immediate post-procedural inflammatory phase that could transiently influence optical reflectance measurements.

VISIA-based quantitative analyses have been widely applied in Asian populations, including studies involving Fitzpatrick skin types III–V treated with picosecond laser systems [5, 25, 33, 36, 37], supporting applicability in comparable ethnic and treatment contexts.

For FocuSkin® measurements (moisture and elasticity), evaluations were performed after a 20-minute acclimatization period in a temperature- and humidity-controlled environment. Triplicate measurements were obtained and averaged to minimize random variation.

### Outcome Framework

The study employed a multidimensional outcome framework incorporating objective instrumental measurements, patient-reported outcomes, physician assessments, and safety parameters.

#### Instrumental Assessments

VISIA® parameters included wrinkles, UV spots, brown spots, texture, red areas, pores, and porphyrins. FocuSkin® parameters included moisture retention and elasticity.

#### Patient-Reported Outcomes

Patient-reported satisfaction was assessed using a 10-item questionnaire scored on a 5-point Likert scale (1–5). Both mean satisfaction scores and the proportion of subjects reporting high satisfaction (score ≥ 4) were analyzed at each visit. The full questionnaire items are presented in Table 3.

#### Clinical Evaluation

Two independent board-certified dermatologists performed PGA scoring on anonymized, coded photographs. Prior to study initiation, a calibration session was conducted to standardize interpretation of the 0–4 scoring scale.

### Safety

Adverse events (AEs) were monitored by telephone follow-up three days after each treatment session and documented using standardized photography.

### Statistical Analysis

This study employed a randomized split-face design in which each subject served as their own control. The left and right cheeks were randomly assigned to either the experimental or control skincare regimen. Repeated assessments were conducted at weeks 4, 8, and 12.

#### Sample Size Estimation

Sample size estimation was based on the expected difference in patient-reported satisfaction ratio (score ≥ 4) at week 12 between the experimental and control sides. Assuming a satisfaction rate of 80% versus 60%, with a two-sided *α* = 0.05 and power (1–*β*) = 80%, at least 25 evaluable subjects were required. Allowing for a 10% dropout rate, 35 subjects were enrolled.

At the time of study planning, reliable reference data for effect size estimation of VISIA-based parameters in a picosecond laser split-face design were not available. Therefore, satisfaction ratio was selected for conservative sample size estimation. Objective instrumental outcomes were analyzed independently using appropriate statistical models.

#### Statistical Methods

Continuous variables are presented as mean ± standard deviation. Within-subject comparisons between treatment sides were initially explored using paired t tests.

Generalized linear mixed models (GLMMs) were applied to instrumental and physician assessment outcomes to account for repeated measurements and within-subject correlation. Models included subject-level random effects and were adjusted for baseline values, age, sex, and visit time. Effect estimates with 95% confidence intervals were reported. Inter-rater reliability for PGA scores was assessed using intraclass correlation coefficients (ICC) based on a two-way random-effects model for absolute agreement.

Patient satisfaction outcomes were analyzed using paired comparisons for both mean Likert scores and the proportion of subjects reporting high satisfaction (score ≥4).

All analyses were performed using SAS version 9.4 (SAS Institute, Cary, NC, USA). A two-sided p value < 0.05 was considered statistically significant.

## Results

### Baseline Characteristics

A total of 35 subjects were enrolled in the study, including 30 females and 5 males, with an average age of 38.97 ± 10.77 years old (range 25–63). Patients aged 25–45 years old comprised 71.4% and those aged ≧ 46 years old comprised 28.6% (Table 1). Only 1 subject did not participate in the 4^th^ visit. There were no significant differences between experimental side and control side cheeks at baseline after the randomized allocation (Table 2).

**Table 1:** Demographic characteristics

Characteristics	N (%)
Sample size	35
Sex	
Female	30 (85.7%)
Male	5 (14.3%)
Age, mean ± SD (min~max)	38.97 ± 10.77 (25 – 63)
Age	
25–45	25 (71.4%)
≥46	10 (28.6%)
Skin Type	
II	14 (40.0%)
III	17 (48.6%)
IV	4 (11.4%)

**Table 2:** Skin features: scores (baselines, visit 1, N=35)

Device	Characteristics	Control side	Experimental side	Paired *t* test
		Mean ± SD	Mean ± SD	*P* value
VISIA	Wrinkles	16.35 ± 11.40	13.69 ±12.35	0.1854
	Texture	8.61 ± 6.94	8.11 ± 7.18	0.6728
	UV spots	19.79 ± 7.90	19.76 ± 8.74	0.9757
	Brown spots	39.95 ± 7.73	39.81 ± 8.44	0.8098
	Pores	13.57 ± 8.09	13.55 ± 8.13	0.9603
	Porphyrin	7.55 ± 9.60	7.53 ± 8.62	0.9607
	Red areas	25.53 ± 5.06	25.19 ± 4.65	0.4189
FocuSkin	Moisture	34.89 ±8.28	36.43 ± 9.39	0.0990
	Elasticity	40.25 ±9.71	43.82 ± 10.3	0.1552

### Effects of 755-nm Picosecond Alexandrite Laser Alone

After adjusting for interference variables of VISIA and FocuSkin evaluation results, laser treatment alone significantly improved pores (p=0.0011), brown spots (p=0.0003), and moisture (p=0.0001) (Supplementary Table 1).

### Effects of Integrated Skincare Adjunct to Laser Treatment

**Fig. 2:**
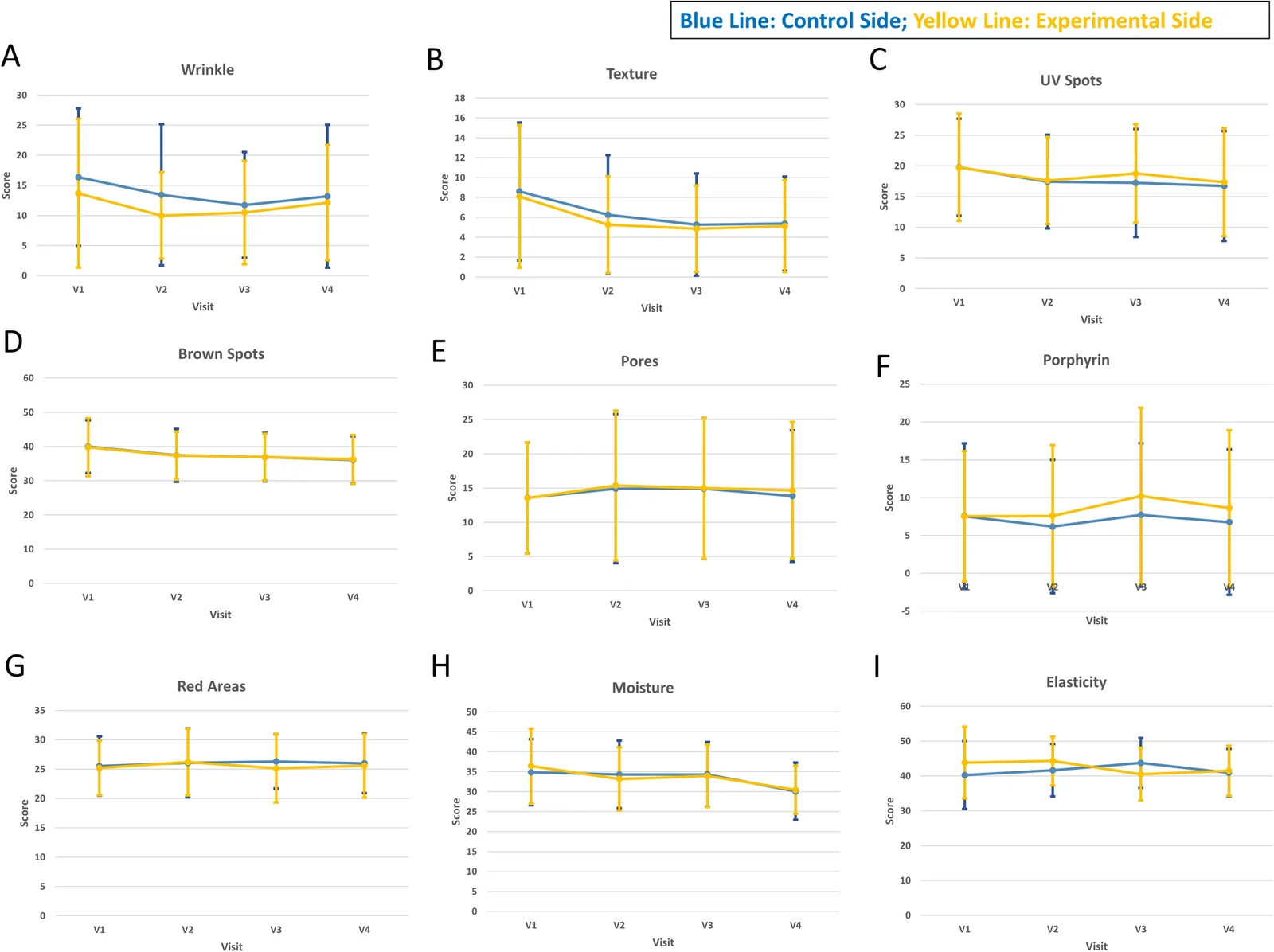
Objective evaluation of skin characteristic scores between control side cheeks (treated with a 755-nm alexandrite laser with DLA treatment alone) vs treatment side cheeks (laser treatments combined with integrated skincare). Wrinkle, texture UV spot, brown spot, pores, porphyrin, and red areas were evaluated by VISIA^®^ system (**A**–**G**). Moisture and elasticity were evaluated by FocuSkin^®^ system (**H**–**I**)

Without adjustment for age or sex, the treatment side demonstrated significantly greater

porphyrin at visits 2, 3, and 4 and significantly improved elasticity at visit 3, as evaluated by VISIA® and FocuSkin® (Fig. 2).

### Adjusted Comparison Between Integrated Skincare and Control

Treatment effects were further evaluated using GLMMs adjusted for age, sex, and visit number. The integrated skincare regimen demonstrated significantly greater improvement compared with the control side for selected VISIA parameters.

Specifically, wrinkle scores showed a significantly greater reduction on the treatment side (adjusted mean difference − 2.33; 95% CI − 4.25 to − 0.41; p = 0.018). Texture scores were also significantly improved on the treatment side (adjusted mean difference − 0.63; 95% CI − 1.18 to − 0.08; p = 0.027).

**Fig. 3:**
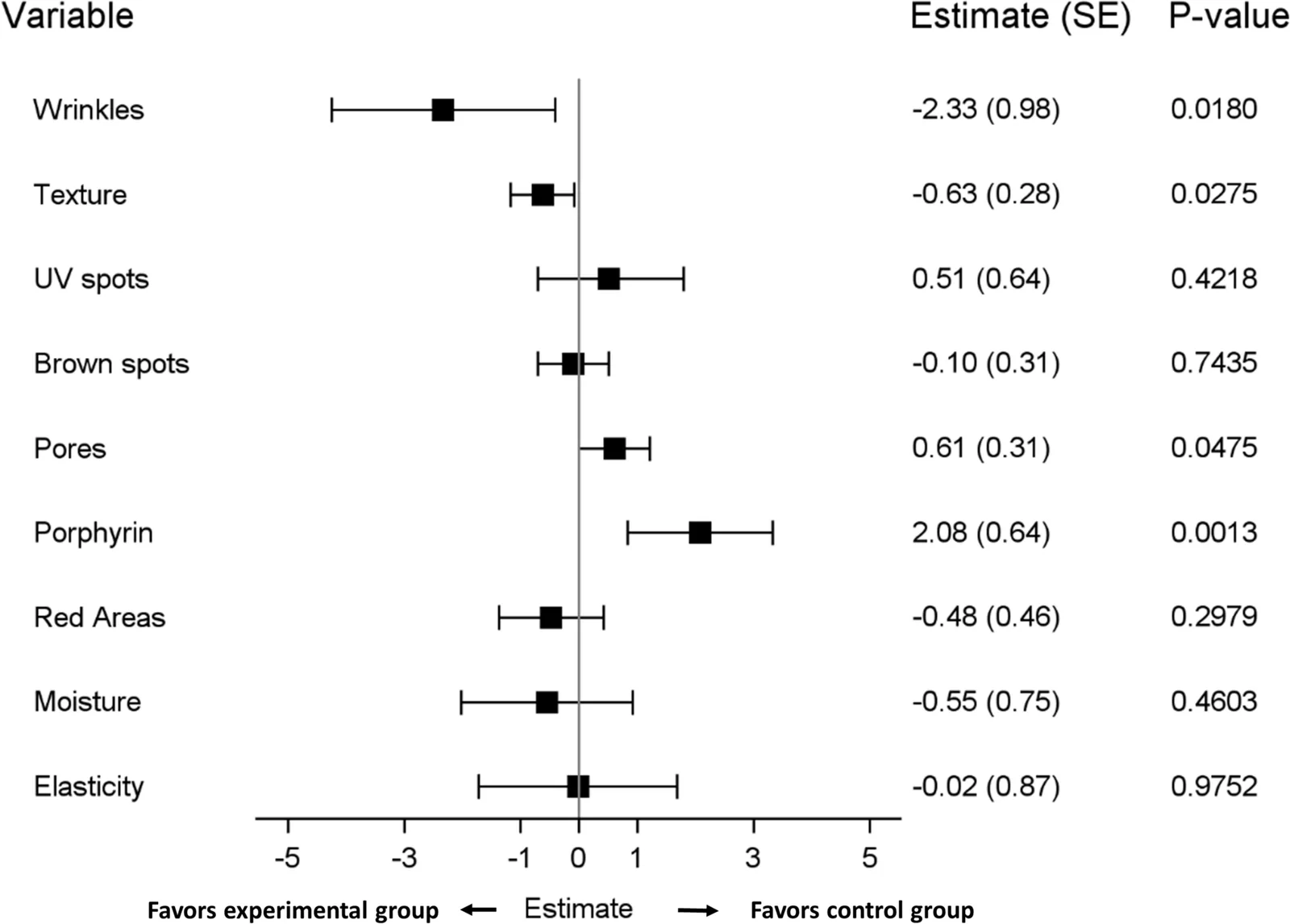
Coefficient plot of generalized linear mixed models comparing skin improvements between control and treatment side cheeks. Generalized linear mixed models were used to assess the characteristic scores with adjustment for baseline, age, sex, and visit time. There were significant improvements in wrinkles (p=0.018) and texture (p=0.028) on the experimental side cheek compared to the control side cheek

**Fig. 4:**
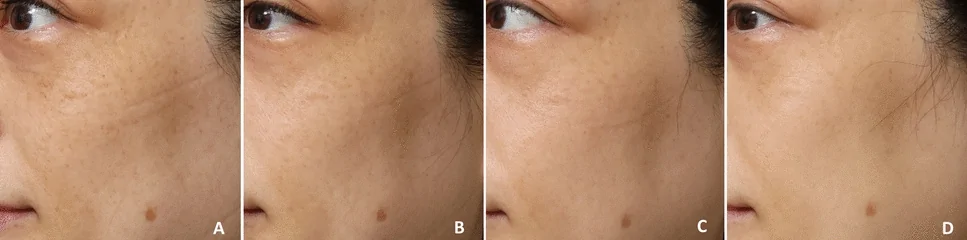
Wrinkles and texture significantly improved after laser and integrated skincare. This 51-year-old lady demonstrated significant improvement after 3 laser treatments and integrated skincare on her left cheek (treatment side). Wrinkle score of VISIA: week 0: 19.073, week 4: 9.341, week 8: 18.816, week 12: 13.673. Texture score of VISIA: week 0: 9.207, week 4: 5.257, week 8: 5.398, week 12: 3.813

**Fig. 5:**
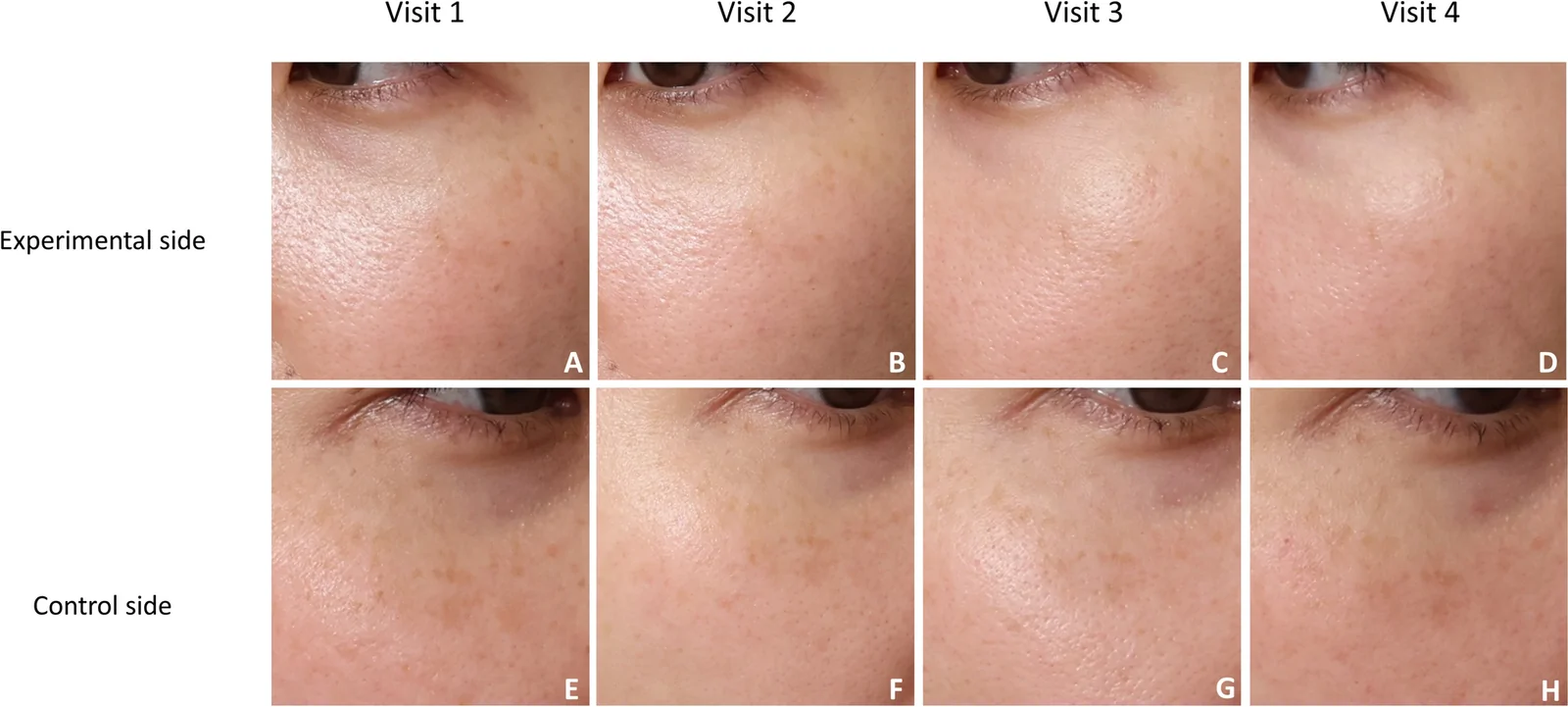
Compare the improvement of wrinkles and texture on the treatment side cheek to the control side cheek. The 33-year-old lady demonstrated greater improvement on the treatment side cheek (left cheek) compared to the control side cheek (right cheek)

For other VISIA parameters and FocuSkin measurements, adjusted differences between sides were not statistically significant. The estimated treatment effects with corresponding 95% confidence intervals are illustrated in Figs.3, 4 and 5.

### Physician’s Global Assessment (PGA) Score

Inter-rater agreement for PGA scores demonstrated good reliability (ICC = 0.82, 95% CI 0.74–0.89). After GLMM with adjustment for age, sex, visit time, and physicians, there was no significant difference between the experimental and control sides (Supplementary Fig 1.)

### Participant’s Overall Satisfaction

Mean satisfaction scores and the proportion of subjects reporting high satisfaction (score ≥ 4) were consistently higher on the experimental side across all visits. Significant between-side differences were observed for most questionnaire domains, particularly those related to hydration, pigmentation improvement, and skin radiance (Table 3; Supplementary Table 1). Total satisfaction scores were significantly greater on the experimental side at all time points (all p < 0.0001).

**Table 3:** Satisfaction score for each visit

Satisfaction	Control side			Experimental side			*P* value		
	Visit 2	Visit 3	Visit 4	Visit 2	Visit 3	Visit 4	Visit 2	Visit 3	Visit 4
1. Skin feels comfortable when using care products.	4.26	4.37	4.29	4.51	4.66	4.65	0.0594	0.0102	0.0002
2. Your skin becomes more hydrated	3.54	3.83	3.82	4.34	4.51	4.56	< 0.0001	< 0.0001	< 0.0001
3. Your skin tone becomes more even.	3.63	3.71	3.79	4.2	4.29	4.41	< 0.0001	< 0.0001	< 0.0001
4. Effective relief for stubborn facial pigmentation.	3.26	3.37	3.59	3.89	3.89	4.26	< 0.0001	< 0.0001	< 0.0001
5. Effectively improves skin discoloration and dullness.	3.51	3.66	3.88	4.03	4.14	4.38	0.0007	< 0.0001	< 0.0001
6. Your skin becomes more delicate	3.89	3.94	3.91	4.46	4.49	4.56	0.0001	< 0.0001	< 0.0001
7. Your skin becomes more radiant.	3.71	3.83	4.12	4.37	4.54	4.56	< 0.0001	< 0.0001	< 0.0001
8. Your skin oil is regulated	3.29	3.49	3.68	3.57	3.86	4.06	0.0161	0.0135	0.0008
9. Your skin’s barrier function is enhanced.	3.54	3.83	3.82	3.94	4.14	4.26	0.0028	0.0013	0.0002
10. Are you satisfied with the overall results of your post-surgical care?	4.03	4.09	4.38	4.46	4.57	4.59	0.0005	0.0008	0.07
Total score	36.66	38.11	39.29	41.77	43.09	44.29	< 0.0001	< 0.0001	< 0.0001

### Safety and Adverse Effects

**Fig. 6:**
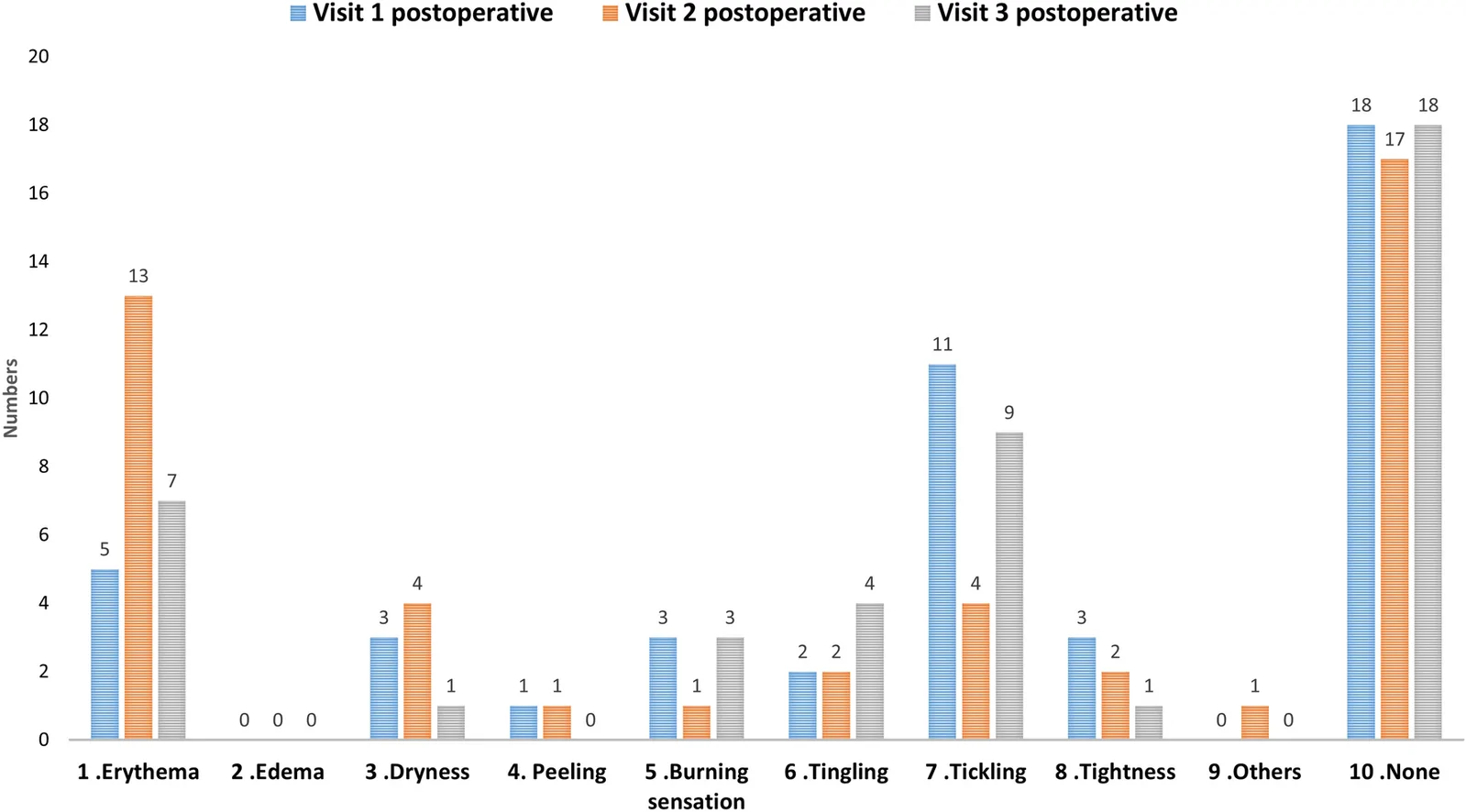
Side effect profile. Erythema and tickling were most commonly reported on Day 3 after the first and third laser treatments

More than half of the patients reported no adverse effects through the study. Transient erythema, regardless of treatment side or control side of cheeks, was the most commonly reported adverse effect (13/35, 37.14%) among all visit time points (Fig. 6).

### Subgroup Evaluation

Patients with the most severe 1/3 UV spot (n = 11, age 25–63, mean age 46.55  ± 11.66) showed significantly better wrinkle improvement on the treatment side of the cheek compared to the control side (p = 0.0313) (Supplementary Fig. 1).

Similarly, patients with the most severe 1/3 brown spot (n = 11, age 28–63, mean age 48.82 ± 11.54) showed significantly better wrinkle improvement on the treatment side of the cheek compared to the control side (p = 0.0037) (Supplementary Fig. 2).

In contrast, patients with less severe UV spots (with lower 2/3 score) (n = 24, age 28–61, mean age 35.50 ± 8.52) showed significantly better texture improvement on the treatment side of the cheek compared to the control side (p = 0.0259) (Supplementary Fig. 3).

**Fig. 7:**
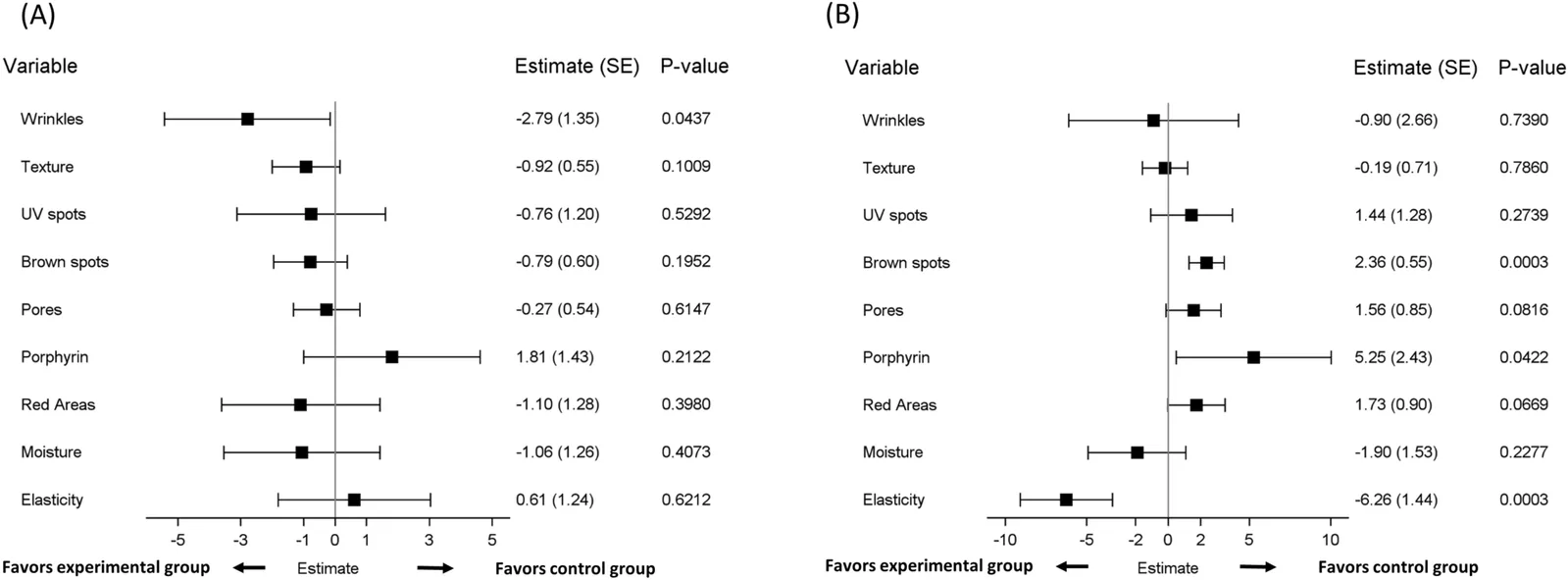
Subgroup analysis. **A** Women over 46 years of age showed significantly greater wrinkle improvement on the treatment side compared with the control side (p = 0.0437). **B** Male patients showed significantly greater elasticity improvement on the treatment side compared with the control side (p = 0.0003)

Female patients over the age of 46 (n = 10, mean age 54.1 ± 5.17 years) showed significantly greater wrinkle improvement (Fig. 7A), while male patients (n = 5, age 30–43 years, mean age 34.4 ± 5.55 years) showed significantly greater elasticity improvement on the treatment side of the cheek compared with the control side (p = 0.0003) (Fig. 7B).

Generalized linear mixed models (GLMMs) were used to assess the characteristic scores with adjustment for confounding factors in the subgroup analysis.

## Discussion

In this prospective, randomized, split‐face study, we evaluated the efficacy and safety of a 755-nm picosecond alexandrite laser with diffractive lens array (DLA) for photoaging, both with and without adjunctive skincare. We demonstrate that laser treatment alone significantly improved pores, brown spots, and skin moisture. When paired with an integrated topical regimen—ferulic acid, tranexamic acid, and skin-identical lipids—additional benefits included marked improvements in wrinkle and overall texture. Notably, women over age 46 achieved greater wrinkle reduction, and male participants exhibited enhanced skin elasticity with adjunctive skincare. Patients presenting with severe brown or UV-induced spots experienced pronounced wrinkle improvement with adjunctive skincare.

Facial photoaging manifests differently in Caucasian and dark skin populations [38–40]. Caucasian skin exhibits earlier and more pronounced signs of wrinkles, sagging, and UV-dependent loss of elastin [39, 41–43]. Caucasians also exhibited faster decreases in viable epidermis thickness and dermal papilla volume than Asian subjects [44]. Fractional CO_2_ lasers demonstrated robust improvements in wrinkles, UV spots, and skin tone, with one study reporting statistically significant wrinkle reduction and lentigines improvement after a single treatment [45, 46]. However, they carry a high risk of post-inflammatory hyperpigmentation (PIH) ranging from 40% to 92.3%, prolonged erythema, and longer recovery times in Asian skin. [47–52]. PIH can persist for up to 6 months and significantly impact quality of life. [48, 51]

Energy‐based devices have been applied to treat facial photoaging in Asian patients with distinct profiles of efficacy and safety [36, 53, 54]. Though non-ablative fractional lasers are effective for improving skin texture and reducing pore size with fewer adverse events and shorter downtime, it is less effective for severe photoaging and deep wrinkles compared to ablative treatments [55]. Microneedling fractional radiofrequency, known for its safety profile suitable for both Caucasian and Asian populations, produces notable gains in skin laxity, pore reduction, and wrinkle improvement [56–58]. However, it may involve more pain and extended downtime [59]. The 755-nm picosecond alexandrite laser primarily utilizes photoacoustic effects, with laser-induced breakdown (LIOB) inducing dermal remodeling and increasing collagen, elastic tissue, and mucin production [60–62]. Selective photothermolysis using chromophores reduces the threshold for LIOB production by 20 times [63]. Asians with abundant epidermal melanin are therefore particularly privileged for picosecond lasers with diffractive lens array using lower energy compared to Caucasians [3, 6, 54, 61, 64]. Studies using 755-nm picosecond alexandrite laser with DLA consistently report significant improvements in global photoaging scores, wrinkles, and pigmentation, similar to fractional ablative laser treatments [36, 53, 54, 61]. Our study showed that 755-nm picosecond alexandrite laser with DLA treatment significantly improved signs of photoaging especially pores and moisture. The neocollagenesis of 755-nm picosecond alexandrite laser with DLA not only led to smaller pores but decreased the TEWL [62]. Using Phyto Corrective with soothing and hydrating effects immediately post-laser and repairing the skin barrier with skin-identical lipids further significantly increase moisture in our study.

Pairing cosmeceuticals with energy‐based treatments yields measurable improvements in skin tone compared with using a single modality [65–67]. We found combining laser and integrated skincare further improved wrinkles and texture. The combination enhances their antioxidative and depigmentation effects. FA is a potent plant-derived antioxidant with photoprotective, anti-aging, anti-inflammatory, and antimicrobial activities [16, 68, 69]. It neutralizes free radicals and inhibits the degradation of collagen and elastin, thereby protecting skin integrity and slowing down the formation of fine lines, wrinkles, and pigmentation spots. FA’s effectiveness is further enhanced when combined with microneedling techniques [15] and 755-nm picosecond alexandrite laser with DLA in our study.

Though dark skin types display delayed onset of wrinkles and sagging due to the natural photoprotection from epidermal melanin and enhanced TGF‑*β* signaling to preserve elastin and collagen fiber networks [70], pigmentary changes emerge earlier and more visible [71]. Our study showed that 755-nm picosecond alexandrite laser with DLA treatment significantly improved brown spots. The dermal remodeling effect of 755-nm picosecond alexandrite laser with DLA eliminates pro-melanogenic senescent fibroblast and lighten the so-called aging pigmentation [72]. Among the integrated skincare products, FA, vitamin C and E in CE serum, kojic acid, and TXA in DD serum all reduced melanin synthesis, while niacinamide in DD serum blocks the transfer of melanosomes [73, 74]. CE serum and DD serum have been shown to reduce hyperpigmentation and are well tolerated when used with laser treatment. [20, 75] Patients with severe UV spots, brown spots, and women over 46 experienced better wrinkle improvement with combined skincare and 755-nm picosecond alexandrite laser with DLA. These patients, who suffer from significant elastin and collagen loss, benefit most from combination therapy.

The 755-nm picosecond alexandrite laser with DLA showed low rates of PIH and lower treatment discomfort compared to fractional CO_2_ laser [76]. In Asian patients with Fitzpatrick skin types III-V, it caused mild, temporary adverse effects including erythema (25%), pruritus/scaling (20%), and reversible hyperpigmentation (5%). [36, 37] However, transient erythema was noted as high as after 95.3% of treatments in one report [5]. In our study, more than half of the patients reported no adverse effects through the study period [22]. Phyto Corrective, CE serum, and DD serum all exhibit anti-inflammatory properties by inhibiting pro-inflammatory cytokines (e.g., IL-1*β*, IL-6, TNF-*α*, arachidonic acid, prostaglandins) and reducing oxidative stress [77–79]. These products have soothing and relieving effects and are particular suitable for post-laser sensitive skincare. In our study, patients were more satisfied with the integrated skincare side than the control side. They reported that their skin felt more delicate, radiant, and hydrated and that using care products felt more comfortable. In the past, medical aesthetic treatments primarily depended on physicians’ recommendations, and women of color often felt underrepresented in the available information on skin aging prevention [2]. In a study where individuals were asked about their skincare needs and barriers to using dermatologic products, respondents of color more frequently cited a lack of available products for their skin type as a barrier compared to their White counterparts [80]. In our study, patients’ active participation and motivation, especially in post-care routines, led to better results.

After accounting for confounding factors, male patients exhibited significantly greater improvements in skin elasticity with combination treatment compared to laser treatment alone. Sex-specific differences in the decline of skin elasticity during aging are attributed to hormonal influences, collagen dynamics, and structural changes in skin layers. Testosterone in males supports thicker skin (25% greater) and slower collagen degradation, while female skin shows more fragmented networks and decreases 20-40% faster after age 50 [81, 82]. However, males experience faster elastic fiber degradation [82, 83. Significant elasticity reduction in women begins in perimenopause, accelerating post-menopause, causing wrinkle length to increase 40% more rapidly than in males [84]. These factors explain why female skin stiffens with age despite greater collagen loss, while male skin maintains thickness but loses recoil capacity later. Our results also explain why female over 46 had better wrinkle improvement, and male had better elasticity improvement from combining laser and integrated skincare. The interplay between hormonal shifts and extracellular matrix remodeling creates distinct trajectories of age-related elasticity decline. [81, 82]

This study has several limitations, including its single-center design and the absence of histologic or molecular validation. Although a split-face design reduces inter-subject variability and enhances statistical efficiency, the potential for minimal systemic absorption or crossover effects from topical skincare products cannot be entirely excluded. Given the localized application area and absence of systemic adverse events, any such effect would be expected to bias results toward the null rather than inflate efficacy estimates.

## Conclusion

The 755-nm picosecond alexandrite laser with DLA improves photoaging and enhances skin quality especially pores, brown spots, and moisture. Combined with integrated skincare products (CE/DD/242), it showed synergistic effect with minimal side effects, further improving wrinkles and texture. With combined treatment, patients with severe brown spots and UV spots, women over 46, saw the most wrinkle improvement, while men experienced the greatest elasticity enhancement.

## Supplementary Information

Below is the link to the electronic supplementary material.

Supplementary file1 (TIF 1192 KB). Patients with the most severe 1/3 UV spots had significantly better wrinkle improvement with adjunctive integrated skin care than laser treatment alone.

Supplementary file2 (TIF 1139 KB). Patients with the most severe 1/3 brown spots had significantly better wrinkle improvement with adjunctive integrated skin care than laser treatment alone.

Supplementary file3 (TIF 1171 KB). Patients with less severe UV spots (with lower 2/3 score) showed significantly better texture improvement with adjunctive integrated skin care than laser treatment alone.

Supplementary file4 (DOCX 16 KB)

## Data Availability

The data supporting the findings of this study are available in this article and the supplementary material.
