# Supplementary material for: Integrated Topical Skincare Enhances Picosecond Laser Treatment Outcomes for Photoaging: A Prospective Split-Face Trial
Source: Aesthetic Plast Surg. 2026 Jul 23;50(17):7401–14. doi: 10.1007/s00266-026-05993-y (PMC13630823; doi:10.1007/s00266-026-05993-y)
Supplement: Supplementary file 1 [file 266_2026_5993_MOESM4_ESM.docx]

Supplementary Table 1 Satisfaction score for each visit

| Satisfaction | Control Side | | | Experimental Side | | | *P*-value | | |
| --- | --- | --- | --- | --- | --- | --- | --- | --- | --- |
|  | Visit 2 | Visit 3 | Visit 4 | Visit 2 | Visit 3 | Visit 4 | Visit 2 | Visit 3 | Visit 4 |
| 1. Skin feels comfortable when using care  products. | 80% | 94% | 94% | 89% | 97% | 100% | 0.2628 | 0.5712 | 0.1604 |
| 2. Your skin becomes more hydrated | 51% | 66% | 76% | 86% | 94% | 100% | 0.0002 | 0.0008 | 0.0031 |
| 3. Your skin tone becomes more even. | 49% | 57% | 65% | 86% | 91% | 94% | <0.0001 | 0.0002 | 0.0008 |
| 4. Effective relief for stubborn facial  pigmentation. | 34% | 34% | 53% | 69% | 69% | 88% | 0.0002 | 0.0002 | 0.0002 |
| 5. Effectively improves skin discoloration  and dullness. | 43% | 54% | 74% | 77% | 86% | 97% | 0.0002 | 0.0004 | 0.0031 |
| 6. Your skin becomes more delicate | 66% | 69% | 76% | 94% | 97% | 100% | 0.0008 | 0.0008 | 0.0031 |
| 7. Your skin becomes more radiant. | 54% | 66% | 88% | 89% | 97% | 100% | 0.0002 | 0.0004 | 0.0437 |
| 8. Your skin oil is regulated | 34% | 34% | 56% | 49% | 60% | 76% | 0.023 | 0.0104 | 0.0062 |
| 9. Your skin's barrier function is  enhanced. | 51% | 60% | 74% | 74% | 83% | 88% | 0.0093 | 0.0032 | 0.023 |
| 10. Are you satisfied with the overall  results of your post-surgical care? | 77% | 86% | 94% | 97% | 100% | 97% | 0.0062 | 0.023 | 0.5715 |
